# Nutritional Risk of Candidates for Simultaneous Pancreatic–Kidney Transplantation—A Narrative Review

**DOI:** 10.3390/nu15194179

**Published:** 2023-09-27

**Authors:** Agnieszka Mizerska, Marek Durlik, Karolina Kędzierska-Kapuza

**Affiliations:** 1 Department of Gastroenterological Surgery and Transplantology, National Medical Institute of the Ministry of Interior Affairs and Administration, Wołoska St. 137, 02-507 Warsaw, Poland; mdurlik@post.pl; 2 Department of Gastroenterological Surgery and Transplantology, Centre of Postgraduate Medical Education, Marymoncka St. 99/103, 01-813 Warsaw, Poland

**Keywords:** simultaneous pancreas and kidney transplantation (SPKTX), pancreatic transplantation, solid organ transplantation, nutritional risk, nutritional intervention, malnutrition, frailty

## Abstract

Introduction: Not much is known about the significance of nutritional status and support in transplant surgery, least of all in simultaneous pancreatic and kidney transplantation. Malnutrition in the context of simultaneous pancreatic–kidney transplantation seems to be complex and a still poorly investigated problem. Since SPKTX is highly qualified and also has a small volume procedure, it is difficult to obtain data from large cohorts of patients. The aim of this article is to gather existing evidence and information about the subject, as well as to elicit some questions and goals for the future. Methods: We searched through the Pub-Med database using the keywords “pancreas and kidney transplantation” combined with “nutritional risk”, “nutritional status”, “malnutrition”, “nutritional intervention”, and “frailty”, finding a total of 4103 matching results. We then narrowed it down to articles written in English with the full text available. We also researched through the references of articles most accurately matching our researched terms. Results: There are numerous tools that have been investigated for the screening of malnutrition, such as the NRI index, PNI index, NLR, SGA scale, and NRS-2002 scale, each of which proved to be of some use in predicting patient outcomes in different surgical settings. Since all of them differed in components and assessed parameters and, in the absence of more sensitive or infallible indicators, the most reasonable approach seems to evaluate them jointly. Conclusion: It is important to underline the necessity of nutritional screening and the subsequent introduction of adequate therapy while awaiting transplantation in an attempt to improve results. Considering the complexity of surgical procedures and the severity of underlying diseases with their intense metabolic components, the patient’s nutritional status seems to significantly influence results. Consequently, nutritional risk assessments should be a part of the routine care of patients qualified for transplantation.

## 1. Introduction

Assessing nutritional risk and the subsequent implementation of nutritional support has a well-established place in the peri-operative care of patients undergoing major gastrointestinal surgery [1]. There are also many studies on the nutritional care of patients with cancer diseases, as well as guidelines based on them [2]. Not much has been said, however, about the significance of nutritional status and support in transplant surgery, least of all in simultaneous pancreatic and kidney transplantation (SPKTX). The aim of this article is to gather existing evidence and information about the subject, as well as to elicit some questions and goals for the future.

It has been largely proved that malnutrition is a risk factor for complications in abdominal surgery. It compromises the healing of anastomoses and surgical incisions on the abdominal wall. Malnourished patients are at risk of increased postoperative morbidity and mortality, lengthened hospital stays, and impaired wound healing [3,4].

There is also evidence of malnutrition compromising the outcomes of transplantation of solid organs, such as the liver, lungs, or kidney [5,6,7,8,9].

The prevailing indication for SPKTX is autoimmune diabetes followed by end-stage diabetic nephropathy, with special consideration for brittle diabetes, resulting in hypoglycemia unawareness, frequent severe metabolic complications, or the failure of exogenous therapy to prevent metabolic complications [10,11]. Also, type 2 diabetes might be an indication, alongside other types, such as diabetes correlated with chronic pancreatitis or post-pancreatectomy cases [12].

There is evidence that SPKTX is more beneficial for these patients than a kidney transplant alone, presumably thanks to better metabolic control [13,14,15]. Nevertheless, this complicated surgical procedure still bears a high incidence of postoperative complications, therefore demanding better identification of potential risk factors and ways of reducing them [16,17,18].

The complexity of the procedure itself and the severity of underlying diseases leading to transplantation in the first place require special attention when qualifying for this treatment. A risk assessment and ability to predict outcomes and distinguish groups of patients that could benefit most from the procedure compared to those whose risk profile is too high seems to be of the utmost importance. Patients’ functional impairment, most commonly assessed using the Karnofsky scale, has been described as an important factor when predicting the outcomes of treatment in many medical fields, including simultaneous pancreas–kidney transplantation [19].

## 2. Aim

Since SPKTX is a highly qualified and small-volume procedure, the aim of this article is to gather existing evidence and information about this subject as well as to elicit some questions and goals for the future.

## 3. Methods

We searched through the Pub-Med database using the keywords “pancreas and kidney transplantation” combined with “nutritional risk”, “nutritional status”, “malnutrition”, “nutritional intervention”, and “frailty”, finding a total of 4103 matching results. We then narrowed it down to articles written in English with their full text available. We also researched through references of articles most accurately matching our researched terms. The summary of our research, divided into subsections discussing pathophysiology, laboratory markers, risk scales, and nutritional intervention, is as follows.

## 4. Results

### 4.1. Pathophysiology of Malnutrition in Spktx-Transplant Recipients

According to ESPEN (European Society for Clinical Nutrition and Metabolism), malnutrition is “a state resulting from lack of intake or uptake of nutrition that leads to altered body composition (decreased fat-free mass) and body cell mass leading to diminished physical and mental function and impaired clinical outcome from disease” [20].

Different forms of malnutrition can be distinguished according to leading pathophysiological pathways. One of them is disease-related malnutrition with inflammation. Inflammatory response, elicited by disease-specific mechanisms, leads to anorexia, reduced food intake, weight loss, and muscle catabolism [21,22,23,24].

Depending on whether the disease is chronic or acute, the inflammatory response might be milder or stronger. Consecutively, apart from the above-mentioned malnutrition in relation to chronic disease, another form of malnutrition caused by the catabolic state is acute disease- or injury-related ones, and major surgical procedures are considered one of its triggers. Stress metabolic response, consisting of high pro-inflammatory cytokine activity, increased corticosteroid and catecholamine release, and insulin resistance, can lead to fast nutrient storage, which is additionally exacerbated by immobilization and reduced food intake [25].

Therefore, there are many reasons for patients prepared for simultaneous pancreatic–kidney transplantations to have compromised nutritional status.

First of all, chronic kidney disease is proven to induce a state of chronic inflammation through various pathways. The kidneys’ compromised ability to excrete acid leads to excess NH_4_^+^ production, resulting in complement activation [26]. Alterations in gut microbiota caused by impaired protein digestion and excess uric acid provoke injury to the intestinal mucosa and, thus, result in the translocation of toxic metabolites and bacterial endotoxins, stimulating pro-inflammatory cytokine production [27,28]. Last but not least, additional sources of inflammation include both hemo- and peritoneal dialysis [29]. Taking into account increased resting energy expenditure in patients with CKD (chronic kidney disease), secondary to a persistent inflammatory state and multiple co-morbidities such as cardiovascular disease, poorly controlled diabetes, and hyperparathyroidism, patients develop a protein–energy wasting syndrome, resulting in the loss of lean body mass [30,31].

Metabolic acidosis that is present in the state of CKD activates proteolysis by activating the ubiquitin–proteasome system and caspase-3, which causes other organ muscle proteins to degrade [32]. Acidosis also contributes to insulin resistance, growth hormone resistance, and glucocorticoid hipersecretion, each of which induces a protein catabolic state [25].

The mode of renal replacement therapy might contribute to malnutrition not only by inducing inflammation but also through other mechanisms. Two main modalities are peritoneal dialysis (PD) and hemodialysis (HD). There is evidence that patients undergoing HD have greater muscle mass and mid-arm circumference [33]. It is estimated that PD might be a risk factor for malnutrition, occurring in 30–50% of patients diagnosed with it [34]. Additional protein loss and higher levels of uremic toxins might also be contributing factors [35,36]. Table 1 summarizes mentioned above causes of malnutrition in SPKTX patients.

The non-inflammatory mechanisms of malnutrition additionally contribute to its development in CKD patients. Patients with insufficient kidney function (CKD 5 stage) but not yet requiring renal replacement therapy have additional risk factors of decreased protein levels due to dietary limitations [37]. Also worth mentioning is possible protein loss due to proteinuria, which occurs in patients with remaining diuresis.

One of the complications of diabetes is gastroparesis, which is delayed gastric emptying that occurs due to neuronal damage caused by chronic hyperglicemia. The result of this might be early satiety, nausea, and vomiting, all of which additionally impair the patient’s nutritional status [38,39]. According to gathered data, approximately 60% of patients suffering from diabetic or idiopathic gastroparesis consume a calorie-deficient diet [40].

Overweight or obese patients are a group requiring special concern. First of all, elevated BMI (body mass index) is a known risk factor in pancreatic transplant surgery [41,42]. Obese patients are also believed to be at a higher risk of complications in other kinds of surgery; however, conflicting data exists [43,44]. Fat tissue has been proven to be metabolically active and can alter the systemic response to surgical injury. In particular, excessive fat tissue in the form of central obesity was proved to be metabolically active, hence inducing and aggravating inflammatory responses. There is evidence that this activity might be particularly strong in patients with CKD [45,46].

As a result of inadequate protein intake and excessive catabolic state, sarcopenia might be present. It is defined as a loss of skeletal muscle mass, strength, and function, leading to increased mortality and worse outcomes in surgical treatment [47]. Diagnostic criteria for sarcopenia are still being discussed [48]. Sarcopenia might be well associated with obesity and, therefore, even more difficult to detect [49,50].

Sarcopenias exacerbate obesity-associated insulin resistance and impair glycemia [51]. The mediators of this are IL-6, which is secreted by muscles, and FGF-21, secreted by muscles in response to insulin [24,52,53]. Muscle also secretes irisin, promoting beta cell survival [54]. Th3 crosstalk between beta cells and myocytes probably has an effect on pancreas graft survival and function [55]. There is evidence of the detrimental impact of sarcopenia on surgical outcomes in pancreas transplant recipients in clinical settings [56,57,58].

Sarcopenia might also be considered as a surrogate of frailty, which has recently been brought to attention as a risk factor for increased mortality and morbidity [59]. The concept of frailty involves a state of vulnerability and non-resilience with limited reserve capacity in major organ systems [60]. Initially described by geriatricians, it made its way into other medical surroundings, investigated as a risk factor for negative outcomes in many non-elderly subpopulations, including solid organ transplant recipients [61,62].

Although the general concept of frailty seems to be widely accepted, tools for its diagnosis are yet to be established. In the literature, there have been numerous scales developed for assessing patients’ frailty in various conditions [63,64]. Some of them have already been proven to predict the outcomes of organ transplantation [65,66,67,68]. The most commonly used is The Physical Frailty Phenotype, which includes weight loss, exhaustion (fatigue), low physical activity, slowness (reduced gait speed), and weakness (low grip strength) [59].

Due to the persistent increased awareness of the prevalence and importance of frailty in transplant settings, the American Society of Transplantation developed a working group designed to discuss the problem of frailty in accordance with different organ transplantation, including the kidney, liver, heart, and lung [69]. Apart from this, there are studies investigating frailty among candidates for simultaneous kidney and pancreas transplantation, estimating its prevalence and assessing it as a potential risk factor. First of all, frailty is associated with a lower chance of being listed for transplantation and higher mortality while being on a waiting list. It is also associated with negative outcomes in transplantations if it finally occurs, such as surgical complications, delayed graft function, postoperative delirium, poor tolerance of immunosuppression, and eventually higher mortality [70].

### 4.2. Laboratory Markers of Malnutrition

There have been several attempts to establish laboratory markers of malnutrition. One of the first was serum albumin, a protein produced by hepatocytes, with its main function as a carrier molecule for the maintenance of the oncotic pressure of blood. Due to its long half-life time (14–20 days) and its distribution along fluid compartments (with more than half of its total amount being located extravascularly), daily protein intake did not significantly influence its levels [71]. It has been proved that albumin levels remain normal during starvation up until it reaches extreme points with other physical evidence, therefore showing its usefulness as a marker of non-inflammatory malnutrition [72]. However, albumin, as a negative acute-phase protein that has been proven to decrease in multiple serious health conditions, tends to be used as a marker of gravity in illness and correlated inflammation, thus affecting nutritional status at the same time and probably indicating patients with special nutritional risk [73]. There is evidence of hypoalbuminemia being a risk factor for impaired outcomes after a SPKTX transplant [74].

Other possible serum markers of malnutrition might be prealbumin, which is affected by an inflammatory state as well as albumin; however, it acts as a more reliable indicator of decreased protein intake because of its significantly shorter half-life time and its smaller total amount [75,76,77]. However, its estimation might be impaired in CKD patients since it is degraded by the kidneys [78]. Another candidate for a serum marker of malnutrition is transferrin; however, there are similar limitations with regard to the interpretation of its serum level—it is increased in iron-deficiency status and in renal failure [77].

Apart from visceral proteins, total lymphocyte count has also been assessed as a potential malnutrition marker [79]. However, it might be of no use to elderly patients, and due to the administration of immunosuppressive therapy, it is useless as a monitoring tool for patients after transplantation.

Serum lipoproteins might also be a meaningful indicator of nutritional status. Total cholesterol [80] and non-HDL cholesterol have both shown a paradoxical effect on Coronary Artery Disease (CAD) in CKD patients. Low levels of total cholesterol and non-HDL-C have been connected with malnutrition indices and the higher risk of CAD in these patients. It was shown that in elderly malnourished patients, HDL cholesterol was low and that a balanced dietary pattern is positively related to the HDL-C level, while there is also a strong negative association between the thrifty dietary pattern and HDL-C [81].

### 4.3. Nutritional and Peri-Operative Risk Assessment

There is no clinically established scoring system concerning recipient-related factors that are designed specifically for SPKTX transplantations. Tools designed to predict outcomes, such as P-PASS (Preprocurement Pancreas Suitability Score) and PDRI (Pancreas Donor Risk Index), analyze donor-related factors. However, there are still conflicting results regarding their clinical usefulness [82,83]. There are a few general surgery risk prediction scoring systems that can be used to assess possible outcomes, such as POSSUM (Physiological and Operative Severity Score for the enumeration of Mortality and morbidity), P-POSSUM (Portsmouth modification of POSSUM), MODS (Multiple Organ Dysfunction Score), the Charlson index, ASA (American Society of Anaesthesiologists), and Waterlow score [84]. None of the aforementioned prediction scales involve the assessment of patients’ nutritional status. 

The Nutrition Risk Index (NRI) is a diagnostic tool designed to predict surgical outcomes with correlation to malnutrition using the serum albumin level and comparing a patient’s actual weight with the usual one [85]. Among the interesting predictive tools is the Prognostic Nutritional Index (PNI), with its calculation depending on serum albumin levels and lymphocyte count [86]. It has been proven to be a useful prognostic tool in lung transplantation [87]. Consistent with the inflammatory undermining of malnutrition, it is also worth considering the neutrophil-to-lymphocyte ratio (NLR) as an indirect marker of subclinical inflammation [88]. There is some evidence of its ability to predict values in kidney transplantation [89]. Widely used screening scales include the Subjective Global Assessment (SGA) [90,91,92] and Nutrition Risk Screening 2002 (NRS-2002), assessing nutritional status together with disease severity and additionally taking into account patient’s older age [93].

The comparison of parameters assessed in the aforementioned index and scales is presented in the table below (Table 2).

### 4.4. Nutritional Intervention

Thanks to growing knowledge and evidence of the influence of nutritional status on the results of surgery, much has been said regarding nutritional support as a part of prehabilitation protocols. Prehabilitation, considered a way of improving the patient’s overall health status prior to elective surgery in order to induce the best possible systemic response for injury, consists of exercise, nutrition, and psychosocial components [94].

We can hardly call transplantation either elective or emergency surgery. When considering deceased donation, as is almost exclusively the case in pancreatic transplantation, the exact time of the procedure cannot be perceived for obvious reasons [95]. Therefore, there are limitations in the ability to plan processes of prehabilitation. Despite diabetic and often nephrological scrutiny, it may be impossible to always detect changes in patients’ nutritional status or body composition with the potential to affect transplantation results at a time sufficient enough to interfere. Having said that, the need for constant vigilance and perhaps adjusted supplementation must be a part of routine care.

Early enteral nutrition, described as an enteral feed initiated within 24 h of surgery, is one of the main parts of enhanced recovery after surgery (ERAS) protocols in abdominal surgery, regarding, for example, esophageal, gastric, pancreatic, or bowel surgery [96,97,98,99]. Some protocols for liver and renal transplantation also exist [100,101]. ERAS protocols in post-pancreatic transplantation surgery are yet to be established [102].

The main concern about introducing early enteral nutrition in pancreas transplant patients concerns the presence of high small bowel anastomosis. However, there have not been RCTs evaluating early enteral nutrition in these patients. Retrospective studies indicate the safety of these modes of nutrition, as well as demonstrating better nutritional support and increasing postoperative albumin levels; however, it has to be underlined that there is no clear evidence of benefits in these exact groups of patients [103]. ESPEN guidelines based on existing evidence suggest early enteral nutrition in pancreatic transplant patients [1].

Apart from concerns about the influence of enteral input on the healing of anastomoses, there is also the possibility of limited tolerance toward early enteral nutrition due to intestinal dysfunction and gastroparesis, especially in the early postoperative phase, as pancreatic transplantation consists of severe surgical injury to the abdominal cavity. As already mentioned, diabetic patients are at risk of delayed gastric emptying. Altogether, the decreased motility of the small intestine due to an autonomic nervous system might be present [104]. If limited tolerance occurs, the patient is at risk of inadequate energy and protein intake. Such a situation might require the additional use of parenteral nutrition [93].

It is important to notice that enteral nutrition does not compromise tacrolimus absorption or blood levels [105]. Enteral nutrition has also been proven to decrease the rate of viral infections after liver transplantations [106]. High-fiber diets enriched with *Lactobacillus plantarum* have also been proven to decrease the rate of infections [107]. However, it has to be underlined that these studies concern liver transplant recipients, and the extrapolation of the results on pancreas transplant recipients requires further investigations. 

There are some data concerning immuno-modulatory substrates available, although they are limited, and there are no recommendations regarding this subject [108]. In the literature, there is some evidence of the beneficial impact of parenteral glutamine supplementation on immune function after surgery as it decreases systemic IL-6 production, therefore improving nitrogen balance; however, data are limited and do not concern transplant recipients in particular [109]. There is also interesting evidence from animal experiments on small bowel transplantations indicating that glutamine supplementation might decrease intestinal mucosa permeability, thus diminishing the rate of bacterial translocation [110].

## 5. Conclusions

Malnutrition in the context of simultaneous pancreatic–kidney transplantation seems to be complex and still a poorly investigated problem. Since SPKTX is highly qualified and also has a small volume procedure, it is difficult to obtain data from large cohorts of patients. Considering the complexity of this surgical procedure and the severity of underlying diseases with their intense metabolic component, the patient’s nutritional status seems to significantly influence results. Consequently, nutritional risk assessments should be a part of routine care in patients who qualify for transplantation. There are numerous tools calculating malnutrition, differing in their components and assessed parameters; therefore, the most reasonable approach seems to be joint evaluation. Its usefulness and predictive value in patients undergoing simultaneous pancreas–kidney transplantation requires further investigation.

## Figures and Tables

**Table 1:** Table summarizing causes of malnutrition in SPKTX patients.

Mechanism	Effects	Reference
Inflammatory response	anorexia, reduced food intake, weight loss and muscle catabolism	[21,22,23,24]
Acute disease- or injury-related one	high pro-inflammatory cytokine activity, increased corticosteroid and catecholamine release and insulin resistance	[25]
Chronic kidney disease	excess NH_4_^+^ production resulting in complement activation	[26]
	metabolic acidosis	[32]
Alterations in gut microbiota	injury to intestinal mucosa translocation of toxic metabolites and bacterial endotoxins	[27,28]
Jatrogenic–hemodialysis or peritoneal dialysis complications	inflammation	[29]
	additional protein loss and higher levels of uremic toxins	[33,34,35,36]
Co-morbidity (cardiovascular disease, poorly controlled diabetes, and hyperparathyroidism)	protein-energy wasting syndrome, loss of lean body mass	[30,31]
Dietary limitations	decreased protein level	[37]
Gastroparesis	early satiety, nausea and vomiting	[38,39,40]

**Table 2:** Comparison between anthropometric parameters and biomarkers in various nutritional assessment and screening tools investigated in transplant surgery (adapted from [77]).

		Prognostic Nutritional Index	Nutritional Risk Index	Subjective Global Assessment (SGA)	Nutritional Risk Screening (NRS) 2002
Anthropometric parameters	weight loss	−	−	+	+
	BMI	−	−	−	+
	present weight	−	+	−	−
	usual body weight	−	+	−	−
History and symptoms	food intake/diet history	−	−	+	+
	gastrointestinal function/symptoms	−	−	+	−
	stress level (severity of diagnosis)	−	−	+	+
	primary diagnosis	−	−	+	−
	physical symptoms	−	−	+	−
	functional capacity	−	−	+	−
Biomarkers	albumin	+	+	−	−
	total lymphocyte count	+	−	−	−

## Data Availability

Not applicable.
